# The Correlation between Corneal Topographic Indices and Corneal High Order Aberrations in Keratoconus

**Published:** 2019

**Authors:** Amr MOUNIR, Islam Saad El SAMAN, Mohamed ANBAR

**Affiliations:** 1 Sohag Faculty of Medicine, Ophthalmology Department, Sohag, Egypt

**Keywords:** Keratoconus, Corneal Topographic Indices, Total Root Mean Square, Coma Aberration, Spherical Aberration, Root Mean Square Astigmatism

## Abstract

This study was performed to investigate aberrometric changes of Keratoconus (KC) and its correlation with corneal topographic indices. In a cross sectional study, the researchers included 170 eyes of 138 candidates that were seeking corneal refractive surgery in Sohag refractive center, Sohag, Egypt and had been diagnosed as clinical KC. Patients were divided to mild, moderate, and severe KC. All eyes included in this study were subjected to corneal tomographic evaluation. Corneal aberrometry data was collected from the Sirius topography (Sirius, Costruzione Strumenti Oftalmici, Italy) over a 5-mm diameter. The collected data included Zernike coefficients for corneal aberrations, including total Root Mean Square (RMS), RMS Spherical Aberration (SA), RMS Coma, and RMS astigmatism. The study population was divided to mild, moderate, and severe KC. Mild KC cases included 58 eyes of 46 patients, moderate KC were 64 eyes of 52 patients, and severe KC were 48 eyes of 40 patients. Root mean square total was statistically significant in all groups with a higher p value in moderate KC (P = 0.001) and also was statistically significant when compared in the three groups altogether (P = 0.0001). Coma aberration was statistically significant in mild and moderate KC and when compared between the three groups (P = 0.0001). Root mean square Trefoil aberration was statistically significant only in moderate KC yet was statistically significant when compared in all groups (P = 0.0001). Root mean square astigmatism was statistically significant in mild KC only and when compared in the three groups altogether (P = 0.0001). Spherical aberration was also statistically significant in moderate and severe KC with a P value of < 0.0001 and 0.001, respectively. There was a positive correlation between posterior elevation and RMS values in mild and moderate KC while there was negative or very weak positive correlation in severe KC. There were negative correlations between the thinnest location and RMS values in nearly all variables in the three groups except weak positive correlation with RMS astigmatism in mild KC and with RMS total and coma aberration in severe KC .In conclusion corneal higher order aberrations measured by the Sirius topography system had low to moderate correlation with corneal topographic indices provided by the same device in different grades of KC.

## INTRODUCTION

Keratoconus (KC) is an ectatic non-inflammatory corneal disorder described by gradual thinning of cornea that results in corneal protrusion, irregular astigmatism, and visual diminution [1]. The disease starts early in puberty and gradually increases until the third to fourth decades. The exact cause for the disease is still unknown, however, hereditary, environmental, and biomechanical factors may play a role in its pathogenesis [2]. Also, it was found that patients having KC showed a higher level of inflammatory mediators in their tear film, which may suggest inflammatory etiology [3]. With the progression of the disease, patients may complain of blurring and distortion of vision due to higher order aberrations [4]. Mild, moderate, and advanced KC could be detected using corneal topography, bio-microscopic, retinoscopic and pachymetry findings [5]. Many indices have been used in the diagnosis of KC and forme fruste KC with different topography devices [6]. In Scheimpflug imaging devices, corneal elevation whether in the anterior or posterior corneal surface are evaluated and the hotly debated question is which of the corneal surfaces has higher sensitivity in perceiving very initial stages of KC [7, 8]. 

Keratoconus is associated with significant high levels of ocular and corneal aberrations when compared with normal eyes [9]. Several previous studies have evaluated different corrected and uncorrected levels of High Order Aberrations (HOAs) in KC. In the uncorrected eyes, it has been found that KC HOAs was about 5.5 times higher than their levels in the normal population and vertical coma alone was responsible for about 53% of the different types of HOAs in KC patients [10]. Several authors suggest that these aberration data can be used to classify subgroups of KC and that the common HOA characteristic of these subgroups can be used to select common optical corrections that may be used for the correction [11, 12]. 

In this study, the researchers investigated aberrometric changes of KC and its correlation with corneal topographic indices.

## METHODS

In this cross sectional study, 170 eyes of 138 candidates seeking corneal refractive surgery in Sohag Refractive Center, Sohag, Egypt that had been diagnosed as clinical KC, were included. All patients involved in this study were informed about the aim of the study and signed an informed consent. The study adhered to the Helsinki declaration and the ethical board committee approval of Sohag Faculty of Medicine was obtained.

Inclusion criteria included any patient with KC aged > 18 years and exclusion criteria were the presence of corneal scarring, history of previous corneal surgeries or ocular pathology, history of systemic medications, collagen vascular diseases, corneal dystrophies, pregnancy or lactation. All keratoconic eyes included in this study had been subjected to corneal topography, and corneal aberrometry had been done for patients using Sirius Scheimpflug placido topography (Sirius, Costruzione Strumenti Oftalmici, Italy). Perfect alignment through the visual axis was assured by asking the patient to look at the central fixation light. Patients were asked to blink between examinations to keep the tear film intact. Eye movement of the subject was regularly tracked by the system, and the quality factor was automatically evaluated. 

Flattest keratometry, steepest keratometry, average keratometry (avge k), maximum keratometry, posterior elevation, anterior elevation, and pachymetry were measured by sirius scheimpflug placido topography, toghether with corneal aberrometry data, which were evaluated over a 5-mm diameter. Corneal aberrometry data included Root Mean Square )RMS( coma, RMS trefoil, RMS total high order aberrations, RMS astigmatism, and RMS spherical aberrations. All keratoconic eyes included in this study were classified to three groups according to average K, where mild (group 1) had average of K< 48 diopters (D), moderate (group 2) had average K between 48 and 52 D and severe (group 3) had average of K > 52 D. Corneal topography and corneal aberrometry were evaluated using Sirius Scheimpflug placido topography (Sirius, Costruzione Strumenti Oftalmici, Italy). The Costruzione Strumenti Oftalmici (CSO) topography system analyzes a total of 6144 corneal points of a corneal area comprised in a circular annulus outlined by an inner radius of 0.33 millimeter (mm) and an outer radius of 10 mm in regards to corneal vertex. Mesopic pupil diameter was acquired in a semi dark room with the disc illuminated in a way to bring ambient light intensity to 4.0 lux, as advised by the original manufacturer [13].

Corneal aberrometry data was collected from the sirius over a diameter of 5 mm. The collected data includes Zernike coefficients for the corneal aberrations, including total RMS, RMS Coma, RMS Spherical Aberration (SA), and RMS astigmatism. Data was analyzed using STATA intercooled version 12.1 (Stata Corporation, College Station, TX, USA). Quantitative data was characterized as mean, standard deviation (SD), median and range. Kruskal-Wallis and Mann-Whitney tests were used accordingly. Spearman’s correlation analyses were performed to find the correlation between average k, posterior elevation, and RMS values. P value was considered statistically significant if it was less than 0.05.

## RESULTS

One hundred and seventy eyes of 138 patients were included in this study. Patients were divided to three groups, including mild, moderate, and severe KC. Mild KC cases included 58 eyes of 46 patients, moderate KC were 64 eyes of 52 patients and severe KC were 48 eyes of 40 patients. Of all the previously mentioned groups, 81 (58.7%) were females and 57 (41.3%) were male. Age of the subjects ranged from 10 to 42 years old. The mean ± SD age of mild KC group was slightly higher; 26.08 ± 6.74 years old, yet this difference was statistically insignificant (P = 0.06) (Table 1). Table 2 summarizes baseline characteristics of corneal topographic indices and HOAs in different groups. Mean ± SD of corneal thickness in mild, moderate, and severe KC was 457.1 ± 33.20, 426.36 ± 32.18, and 400.28 ± 37.39.

**Table 1:** Age Characteristics in Different Groups

Variable	Keratoconus		
	Mild (n = 46)	Moderate (n = 52)	Severe (n = 40)
Age/years			
Mean ± SD	26.08 ± 6.74	23.00 ± 5.85	24.43 ± 6.98
Median (range)	24.5 (14-42)	23 (10-41)	23.5 (12-39)
P1 = 0.06, P2 = 0.68, P3 = 0.86			

SD: Standard Deviation; n: Number, P1 compared mild and moderate, P2 compared mild and severe and P3 compared moderate and severe.

**Table 2:** Baseline Characteristics of Corneal Topographic Indices and High Order Aberrations in Different Groups

Variable	Mean ± SD, Median (range)	Mean ± SD, Median (range)	Mean ± SD, Median (range)
	Mild KC	Moderate KC	Severe KC
Average K	45.90 ± 1.40, 46 (42.86-51.91)	49.74 ± 1.30, 49.84 (46.76-51.91)	55.70 ± 3.15, 55.05 (52.25-69.77)
P1<0.0001*, P2<0.0001*, P3<0.0001*			
Posterior elevation	28.47 ± 14.26, 25 (10-75)	42.59 ± 12.97, 39 (20-84)	67.73 ± 19.29, 64 (28-120)
P1=0.0001*, P2=0.0001*, P3=0.0001*			
RMS total	1.05 ± 0.64, 1.0 (0.23-2.39)	1.95 ± 0.81, 1.92 (0.49-4.2)	3.21 ± 1.06, 3.2 (1.1-6.26)
P1=0.0001*, P2=0.0001*, P3=0.0001*			
RMS coma	0.86 ± 0.61, 0.76 (0.1-2.09)	1.69 ± 0.86, 1.72 (0.06-3.93)	2.55 ± 0.97, 2.62 (0.25-4.54)
P1=0.0001*, P2=0.0001*, P3=0.0001*			
RMS Trefoil	0.34 ± 0.19, 0.3 (0.07-0.9)	0.51 ± 0.29, 0.47 (0.09-1.2)	1.01 ± 0.47, 0.96 (0.13-2.83)
P1=0.0007*, P2=0.0001*, P3=0.0001*			
RMS Astigmatism	1.58 ± 0.92, 1.37 (0.21-3.65)	1.97 ± 1.22, 1.86 (0.23-6.02)	3.42 ± 2.11, 3.1 (0.07-10.13)
P1=0.12, P2=0.0001*, P3=0.0001*			
RMS SA	0.18 ± 0.14, 0.15 (0.01-0.58)	0.39 ± 0.31, 0.3 (0.02-1.3)	0.66 ± 0.59, 0.5 (0.04-2.34)
P1=0.0001*, P2=0.0001*, P3=0.01*			

SD: Standard Deviation; RMS: Root Mean Square: SA: Spherical Aberration

* : P values less than 0.05 is Significant. P1 compared Mild and Moderate, P2 compared Mild and Severe and P3 compered Moderate and Severe

According to the statistical analysis of the corneal HOAs in these different grades of KC, this research found a positive correlation between average keratometry and RMS values (Table 3). Correlation of RMS total with average k was positive with statistically significant values in all groups with a higher p value in moderate KC (P = 0.001) and also was statistically significant when compared in the three groups altogether (P = 0.0001). Correlation of RMS total coma with average k was positive with statistically significant values in all groups and it was statistically significant in mild and moderate KC (P = 0.03 in both) and when compared in the three groups altogether (P < 0.0001). Correlation of RMS Trefoil with average k was statistically significant in moderate KC only (P = 0.04) and also statistically significant when compared in all groups (P < 0.0001). Correlation of RMS astigmatism with average k was positive with statistically significant values in mild KC only (P = 0.02) and all RMS values had significant positive correlation with average k when considered in total (P < 0.0001). Correlation of SA with average k was positive with statistically significant values in moderate and severe KC with a P value of less than 0.0001 and 0.001, respectively, and when compared in the three groups altogether (P < 0.0001). (Table 3). With regards to the correlation between posterior elevation and RMS values, the researchers found a positive correlation between posterior elevation and RMS values in mild and moderate KC while there was weak or negligible negative or weakly positive correlation in severe KC. In mild KC all RMS values were statistically significant except for RMS astigmatism (P = 0.17), in contrast to moderate KC, where RMS total was the only statistically significant value (P = 0.03) and in severe KC, RMS SA was statistically significant with a P value of 0.004 while other RMS values were insignificant. Interestingly, all RMS values had significantly weak to moderate positive correlation with posterior elevation when considered in total (Table 4).

**Table 3:** Correlation between Average k and Root Mean Square (RMS) Values.

Variables	Keratoconus							
	Mild		Moderate		Severe		Total	
	**r**	**P**	**r**	**P**	**r**	**P**	**r**	**P**
RMS total	+0.36	0.01*	+0.42	0.001 *	+0.29	0.03 *	+0.78	<0.0001 *
RMS coma	+0.28	0.03 *	+0.27	0.03 *	+0.24	0.08	+0.68	<0.0001 *
RMS Trefoil	+0.17	0.20	+0.26	0.04 *	+0.19	0.16	+0.66	<0.0001 *
RMS Astigmatism	+0.31	0.02 *	+0.18	0.16	+0.11	0.43	+0.45	<0.0001 *
RMS Spherical aberration	+0.01	0.92	+0.57	<0.0001 *	+0.44	0.001 *	+0.53	<0.0001 *

r: correlation co-efficient; P: p-value;

* : p-values less than 0.05 is significant.

**Table 4:** Correlation between Posterior Elevation and Root Mean Square (RMS) Values.

Variables	Keratoconus							
	Mild		Moderate		Severe		Total	
	**r**	**P**	**r**	**P**	**r**	**P**	**r**	**P**
RMS total	+0.58	<0.0001*	+0.26	0.03 *	-0.01	0.92	+0.68	<0.0001 *
RMS coma	+0.56	<0.0001 *	+0.14	0.28	-0.07	0.64	+0.61	<0.0001 *
RMS Trefoil	+0.43	0.001 *	+0.04	0.76	+0.01	0.92	+0.59	<0.0001 *
RMS Astigmatism	+0.18	0.17	+0.24	0.06	+0.03	0.85	+0.43	<0.0001 *
RMS Spherical aberration	+0.31	0.01 *	+0.01	0.92	-0.39	0.004 *	+0.34	<0.0001 *

r: correlation co-efficient; P: p-value; SD: Standard Deviation; RMS: Root Mean Square;

* : p values less than 0.05 is significant.

Concerning the corneal thinnest location, the research found a negligible or low or weakly negative correlation between the thinnest location and RMS values in nearly all variables in the three groups except a weak positive correlation with RMS astigmatism in mild KC (r = +0.13, P = 0.32) and with RMS total and coma aberration in severe KC (r = +0.03, P = 0.81; r=+0.09, P = 0.51). Root mean square total and coma aberration were significantly correlated with thinnest location in both mild and moderate KC groups. However, other variables, including RMS astigmatism, RMS Trefoil and RMS SA had insignificant correlation with thinnest location in three groups. Interestingly, all RMS values had significantly negligible or weak correlation with thinnest location when considered in total (Table 5).

**Table 5:** Correlation between Thinnest Location (CT) and Root Mean Square (RMS) Values.

Variables	Keratoconus							
	Mild		Moderate		Severe		Total	
	**r**	**P**	**r**	**P**	**r**	**P**	**r**	**P**
RMS total	-0.53	<0.0001*	-0.38	0.002 *	+0.03	0.81	-0.60	<0.0001 *
RMS coma	-0.55	<0.0001 *	-0.36	0.003 *	+0.09	0.51	-0.55	<0.0001 *
RMS Trefoil	-0.16	0.21	-0.21	0.09	-0.07	0.62	+0.44	<0.0001 *
RMS Astigmatism	+0.13	0.32	-0.07	0.60	-0.22	0.11	-0.25	0.001 *
RMS Spherical aberration	-0.13	0.33	-0.06	0.63	-0.11	0.42	-0.28	0.0002 *

r: correlation co-efficient; P: p value; SD: Standard Deviation; RMS: Root Mean Square;

* : p values less than 0.05 is significant.

## DISCUSSION

Optical aberrations result in decreased visual quality and distorted images. High order aberrations affect the retinal image quality in patients with irregular cornea [14]. High order aberrations, including total aberration, coma aberration and spherical aberration were deeply evaluated in several previous studies and found to be increased in patients with KC [15-17]. In this study, the aberrometric changes of KC were evaluated, including changes in the HOAs at the anterior corneal surface and the correlation of the corneal topographic indices measured by Sirius Scheimpflug placido topography with these aberrations. This study evaluated the correlation between the corneal HOAs and the most important topographic indices measured by corneal tomography. The results were a positive correlation between posterior elevation and RMS values in mild and moderate KC while there was negligible negative or negligible or weakly positive correlation in severe KC. Also, there was a significant negative correlation between the thinnest location and RMS values in nearly all variables in total, except significant weak positive correlation with RMS trefoil. Additionally, there was a significant correlation between average K and total aberrations (total RMS values). 

Coma, trefoil, astigmatism, and spherical aberrations were found to increase in advanced groups of KC. Colak et al. compared the corneal topographic indices and the anterior high order corneal aberrations in KC and normal eyes by using Scheimpflug-Placido topography, and found that there was a high correlation between corneal curvature and total aberrations [18]. Maeda et al. [19] compared wave front aberrations of normal and keratoconic eyes and evaluated the characteristics of the HOAs in KC eyes measured with Hartmann-Shack sensor. They concluded that the increase of ocular HOAs in keratoconic eyes results from an increase of corneal HOAs.

In the current study with regards to the posterior elevation, this study found significant differences in all variables of HOAs when comparing groups of KC patients in total. However, there was a positive correlation between posterior elevation and RMS values in mild and moderate KC groups while there was a negative or positive correlation in severe KC, which can be explained by the marked difference of changes in HOAs and very high posterior elevations in severe cases of KC. This coincides with the study of Delgado et al., [20] who evaluated the correlation between HOAs in anterior corneal surface and the degree of KC measured with a Scheimpflug camera and found that coma aberration was significantly correlated with KC severity, yet RMS Trefoil and KC were weakly correlated.

 In a study by Nakagawa et al., [21] they investigated corneal Higher-Order Aberrations (HOAs) in keratoconic eyes compared with normal eyes by the rotating camera system (Pentacam; Oculus, Inc.). They found that corneal HOAs on corneal surfaces in eyes with KC were higher than in control eyes, which coincides with the results. All these previous data can be used as a method for staging KC. Alio et al., [12] showed that HOAs of the anterior corneal surface can be used to detect grades of KC by using corneal map analysis video keratoscopy.

Different studies have evaluated optical aberrations in KC eyes and showed increased HOA, especially coma and spherical aberrations, as found in the current study [9], [22]. This significant increase in HOA leads to reduced visual acuity, which cannot be corrected with spectacles or soft contact lenses [23]. Intracorneal ring segments and phakic Toric Implantable Collamer Lens are effective methods in patients with KC based on their reduction effect for optical aberrations [24]. 

## CONCLUSION

Corneal higher order aberrations measured by the Sirius Scheimpflug camera placido corneal topography system were correlated with corneal topographic indices provided by the same device in different grades of KC.
